# In Vitro *α*-Amylase and *α*-Glucosidase Inhibitory Potential of Green Banana Powder Extracts

**DOI:** 10.1155/2024/5515855

**Published:** 2024-09-07

**Authors:** Pongsathorn Klomsakul, Pornchanok Chalopagorn

**Affiliations:** ^1^ Department of Biology Faculty of Science and Technology Phranakhon Rajabhat University, Bangkok 10220, Thailand; ^2^ Department of Chemistry Faculty of Science and Technology Phranakhon Rajabhat University, Bangkok 10220, Thailand

## Abstract

This study investigated the proximate composition and inhibitory potential of hot water and ethanolic extracts of the pulp, peel and whole fruit of green banana (*Musa sapientum*) on *α*-amylase and *α*-glucosidase. Bioactive compounds were identified using GC-MS analysis. In addition, the cytotoxic effect on human gingival fibroblast (hGF) was evaluated using the sulphorhodamine B (SRB) assay. The results showed that the peel of green banana had the highest amount of ash (10.05%), fat (2.83%), protein (3.64%) and total dietary fibre (36.62%). The carbohydrate content of the whole fruit (81.79%) and pulp (81.50%) was higher than that of the peel (71.90%). The moisture content of the pulp (13.08%) was higher than that of the peel (11.58%) and whole fruit (11.30%). The ethanolic green banana peel extract showed a good inhibitory effect of *α*-amylase and *α*-glucosidase with the concentration necessary for 50% inhibition (IC_50_) of 0.512 and 0.100 mg·mL^−1^, respectively. The *α*-glucosidase inhibitory effect of the ethanolic green banana peel extract and the hot water green banana peel extract was not significantly different from that of acarbose (IC_50_ 0.108 mg·mL^−1^). GC-MS analysis of the ethanolic green banana peel extract revealed fatty acids and fatty acid ester (9-octadecenamide (*Z*), octadecanamide and other compounds). The ethanolic peel extract exhibits a significant noncytotoxicity effect on hGF cells at concentrations ranging from 0.0001 to 1.0 mg·mL^−1^.

## 1. Introduction

According to the International Diabetes Federation (IDF), there are more than 530 million diabetic patients globally. This number is increasing rapidly since diabetes mellitus (DM) is one of the most prevalent metabolic disorders worldwide. The condition is closely linked to chronic hyperglycaemia, and it is anticipated that the number of cases will exceed 640 million by 2030, a number that could be more than 780 million by 2045 [1]. DM usually involves inadequate secretion of insulin caused by damage to the *β* cells that produce insulin in the pancreas. Hyperglycaemia follows, which in turn can cause severe problems in the long term, including organ failure in some cases, typically affecting the nerves, kidneys, eyes and heart [2]. The condition has been linked to lifestyle factors such as obesity and a lack of exercise, as well as oxidative stress. DM can be addressed initially through exercise programmes and a healthy eating plan, while subsequent treatments include insulin or drugs designed to reduce the blood sugar levels of patients [3].

One of the therapeutic approaches in diabetes treatment is based on the inhibition of *α*-amylase and *α*-glucosidase activity, since these enzymes are responsible for digesting and absorbing carbohydrates. Type 2 diabetes can be treated by the use of miglitol, acarbose and voglibose, which are *α*-glucosidase inhibitors. The problem, however, is that hypoglycaemic drugs can lead to adverse side effects such as diarrhoea, flatulence, liver disorders and abdominal cramping [4]. As a consequence, natural sources of hypoglycaemic agents have become an important area of research; the aim is to avoid the undesirable side effects of the drugs currently used to treat diabetes as well as to reduce the costs involved.

One of the most widely grown fruits in the world is the banana, which can be found in more than a thousand different varieties. While production is high, more than 30% of the total crop yield typically goes to waste because people have a strong preference for only fully ripened bananas [5, 6]. Consequently, the commercial processing of bananas has been developed and adjusted to minimise this waste, and further research into this issue is ongoing. Waste can be utilised in the form of green banana powder (GBP), which can be obtained from bananas in Stages 1 and 2 on the Von Loesecke maturation scale [7]. These bananas are green, or green with a hint of yellow. GBP is becoming increasingly popular for human consumption since it offers notable benefits in terms of nutritional value and physiological support. It is an excellent source of vitamin C, vitamin B6 and provitamin A and is also high in minerals such as zinc, magnesium, potassium and phosphorus [5, 8]. In addition, it contains resistant starch, commonly used in the food sector since it offers health benefits through the promotion of microbiota in the human gut [9]. As a result, it is used as an ingredient in gluten- and wheat-free food products [10].

As it is rich in numerous bioactive components, GBP is a promising and attractive target for antidiabetes studies. GBP exhibits multifaceted potential by preventing oxidative damage to the liver and kidneys, enhancing the biochemical parameters in type 1 diabetic rats [11], reducing body weight and improving insulin sensitivity in obese type 2 diabetics [12]. Furthermore, it shows promise in ameliorating type 2 diabetes and associated cardiovascular risks [13]. However, the nutritional composition of GBP depends on the soil, climate, banana variety, mutation stage, local production and other factors [5]. The study of various parts of plants has contributed significantly to the discovery of numerous biologically active drugs [14]. Various components of bananas, including the peel, pulp and whole fruit, exhibit dissimilar percentages of phenolic compounds and flavour components [15]. It is widely recognised that various solvents have the capacity to extract distinct phytochemical groups depending on their polarity, consequently leading to variations in the biological activity of the extracts [16]. Hence, the objective of the present study was to investigate the proximate composition and inhibitory potential of hot water and ethanolic extracts of the pulp, peel and whole fruit of green banana (*Musa sapientum*) on *α*-amylase and *α*-glucosidase. In addition, the bioactive compounds can be identified using gas chromatograph interfaced with a mass spectrometer (GC-MS) analysis.

## 2. Materials and Methods

### 2.1. Plant Material

Green bananas (*Musa sapientum*; Musa ABB cv. Kluai “Namwa”) at Maturation Stage 1 were collected in May 2022 from the Chu-Jit banana cultivating farm, Nong Yai To Subarea, Chai Badan Area, Lopburi Province, Thailand. Bananas were identified by the Forest Herbarium, Department of National Park, Wildlife and Plant Conservation, Bangkok, Thailand.

### 2.2. Preparation of GBP

Green bananas were washed with water and then separated into three portions, including the pulp, peel and whole fruit. The samples were cut into small pieces and dried immediately at 50°C for 24 h using a hot-air oven. Subsequently, the dried samples were ground to a fine powder using a mechanical blender.

### 2.3. Proximate Composition

The proximate parameters (ash, moisture, carbohydrate, fats, protein and fibre contents) of GBP were determined using the method suggested by the Association of Official Analytical Chemists (AOAC) (2019) [17]. The food energy values [18] were expressed as

(1) $$\begin{array}{c} \text{food energy }\left(\text{kcal}\right)=\left(\%\text{ protein}\times 4\right)+\left(\%\text{ fat}\times 9\right)+\left(\%\text{ carbohydrate}\times 4\right). \end{array}$$

### 2.4. Extraction of GBP

#### 2.4.1. Hot Water Extraction

Ten grams of each sample (pulp, peel and whole fruit) was extracted in 200 mL of hot water at 80°C for 10 min on a hot plate, followed by maceration with an ultrasonicator for 1 h. The extract was then filtered with a Whatman filter paper (110 mm) and centrifuged at 3500 × *g* for 15 min (Centrifuge 5810 R, Eppendorf AG, Hamburg, Germany). The extract was dried by evaporation under vacuum and stored at 4°C.

#### 2.4.2. Ethanol Extraction

Ten grams of each sample (pulp, peel and whole fruit) was extracted in 200 mL of 80% ethanol by maceration with an ultrasonicator for 1 h. The extract was then filtered with a Whatman filter paper (110 mm) and centrifuged at 3500 × *g* for 15 min (Centrifuge 5810 R, Eppendorf AG, Hamburg, Germany). The extract was dried by evaporation under vacuum and stored at 4°C.

### 2.5. Determination of *α*-Amylase Inhibition

The *α*-amylase inhibition was determined by assay, as previously reported [19]. Porcine pancreatic *α*-amylase was prepared using 0.5 mg·mL^−1^ in a 20 mM sodium phosphate buffer (pH 6.9). The preincubation mixture consisted of 50 *μ*L of *α*-amylase and 50 *μ*L of GBP extract. The mixture was incubated at 25°C for 10 min. Thereafter, 50 *μ*L of 0.5% starch solution in 20 mM sodium phosphate buffer (pH 6.9) was added and incubated at 25°C for 10 min. The reaction was stopped by adding 50 *μ*L of 96 mM 3,5-dinitrosalicylic acid and incubating it in a boiling water bath for 10 min. The absorbance of the solution was then measured spectrophotometrically at 540 nm (VICTOR Nivo, PerkinElmer, Massachusetts, USA). Subsequently, a control was prepared using the same procedure except that the extract was replaced with a buffer. Acarbose was used as a positive control. The extent of inhibition by the addition of samples was expressed as the concentration necessary for 50% inhibition (IC_50_). The percentages of *α*-amylase inhibition were expressed as

(2) $$\begin{array}{c} \%\text{ inhibition}=\left[\frac{A_{\text{control}}-A_{\text{sample}}}{A_{\text{control}}}\right]\times 100. \end{array}$$

### 2.6. Determination of *α*-Glucosidase Inhibition

The *α*-glucosidase inhibition was determined by assay, as previously reported [19, 20]. *α*-Glucosidase from a *Saccharomyces cerevisiae* solution was prepared using 1 U·mL^−1^ in a 20 mM sodium phosphate buffer (pH 6.9). The preincubation mixture consisted of 50 *μ*L of *α*-glucosidase and 25 *μ*L of GBP extract. The mixture was incubated at 25°C for 10 min. Thereafter, 25 *μ*L of 5 mM *p*-nitrophenyl-*α*-D-glucopyranoside solution in 20 mM sodium phosphate buffer (pH 6.9) was added and incubated at 25°C for 5 min. The absorbance was measured at 405 nm using a spectrophotometer (VICTOR Nivo, PerkinElmer, Massachusetts, USA). Subsequently, a control was prepared using the same procedure except that the extract was replaced with a buffer. Acarbose was used as a positive control. The extent of inhibition by the addition of samples was expressed as the concentration necessary for IC_50_. The percentages of *α*-glucosidase inhibition were expressed as

(3) $$\begin{array}{c} \%\text{ inhibition}=\left[\frac{A_{\text{control}}-A_{\text{sample}}}{A_{\text{control}}}\right]\times 100. \end{array}$$

### 2.7. GC-MS Analysis

In this study, gas chromatography and mass spectrometry were used to identify the phytochemical compounds present in the ethanol extract of green banana peel. GC-MS analysis was carried out on a GC Shimadzu QP2020 and GC-MS equipped with a SH-Rxi-5Sil MS capillary column (30 m × 0.25 mm ID × 0.25 *μ*m film thickness, Shimadzu, Japan). Helium (99.99%) was the carrier gas at a constant flow rate of 3 mL·min^−1^. The oven temperature programme was 50°C (isothermal for 2 min), with an increase of 10°C·min^−1^ to 280°C for 3 min. The total GC-MS running time was 28 min. The relative percentage of each component was evaluated by comparing its average peak area to the total area.

### 2.8. Determination of Cytotoxic Activity

For the sulphorhodamine B (SRB) assay, human gingival fibroblast (hGF) cells were seeded onto 96-well plates and treated with different concentrations of the GBP peel extract at 0.0001, 0.001, 0.01, 0.1 and 1.0 mg·mL^−1^ for 48 h. Cells were fixed by adding 100 *μ*L of cold 10% trichloroacetic acid for 1 h at 4°C. Thereafter, the supernatant was removed, and cells were rinsed four times with slow-running tap water. The plate was allowed to air-dry and stained with 100 *μ*L of 0.057% SRB solution at room temperature for 30 min. Subsequently, the wells were washed four times with 1% (v/v) acetic acid to remove unbound dye. The bound SRB was dissolved by 100 *μ*L of 10 *μ*M Tris, pH 10.5, for 10 min. The absorbance was measured at 510 nm (VICTOR Nivo, PerkinElmer, Massachusetts, USA). Cells treated with a culture medium served as the negative control, while those treated with sodium lauryl sulphate (SLS) were the positive control [21].

### 2.9. Statistical Analysis

Comparison of the means of three replicates was carried out for mean ± standard deviation to establish whether there were differences between the activities of samples, and the variance analysis was applied to the result. Values of *p* ≤ 0.05 were considered significantly different (*α* = 0.05).

## 3. Results and Discussion

### 3.1. Proximate Composition

The ash, moisture, carbohydrate, fat, protein and fibre contents in different parts of GBP in g/100 g dry weight are shown in Table 1. GBP peel had the highest amount of ash (10.05%), fat (2.83%), protein (3.64%) and total dietary fibre (36.62%). The carbohydrate content of GBP pulp (81.50%) and whole fruit (81.79%) did not reveal a significant difference, though it was higher than that in the peel (71.90%) with a significant difference. The moisture content of the peel (11.58%) and whole fruit (11.30%) was less than that of the pulp (13.08%) with a statistically significant difference. The results revealed that GBP is a good source of healthy minerals, nutrients and dietary fibre. Ash content in foods refers to the amount of minerals in food [18]. This indicates that GBP peel is rich in minerals. Fat, protein and carbohydrate contents provide energy for the body. The energy value of GBP was ranked in the order of whole fruit (345.01 kcal) > pulp (339.12 kcal) > peel (327.63 kcal). Variances in protein and fat contents may be due to geographical location and varietal differences [5, 18]. Total dietary fibre has various health benefits, including the prevention of heart disease, hypertension, diabetes, obesity and gastrointestinal diseases [22]. The total dietary fibre content in GBP peel was found to be approximately three times higher than that in the pulp and whole fruit. Moisture content in food products influences their shelf life. The moisture in flour should be at an acceptable limit of not more than 10% for long-term storage [23]. The moisture content of GBP pulp, peel and whole fruit was found to be from 11.30 to 13.08%, which is not suitable for storage stability and longer shelf life.

### 3.2. Inhibition of *α*-Amylase

The percentage of *α*-amylase inhibition of hot water or ethanolic extracts of different parts of GBP is presented in Table 2. The results revealed that the *α*-amylase inhibition of GBP extracts increases slightly as the concentration increases. Acarbose had the highest inhibitory *α*-amylase activity at all concentrations. At 2.0 mg·mL^−1^, the pulp, peel and whole fruit of hot water extract did not show a significant difference in the *α*-amylase inhibition (Table 2). However, there were significant differences in the inhibition rates of ethanol extracts (Table 3). The *α*-amylase activity of the GBP extracts is summarised in Table 4. All GBP extracts had a higher value of IC_50_ than acarbose as the positive control. IC_50_ values of extracts for *α*-amylase activity ranged from 0.512 to 1.615 mg·mL^−1^, while the IC_50_ value of acarbose was 0.219 mg·mL^−1^. Previous reports have shown that severe inhibition of pancreatic *α*-amylase may lead to aberrant bacterial fermentation of undigested starch in the colon. Thus, modest *α*-amylase inhibitory action appears beneficial [24].

### 3.3. Inhibition of *α*-Glucosidase

The results of *α*-glucosidase inhibition for hot water and ethanolic extracts of different parts of GBP showed that 0.05 to 0.5 mg·mL^−1^ concentrations of pulp, peel and whole fruit extracts were significantly different among the *α*-glucosidase inhibition values for all extracts (Tables 5 and 6). All extracts showed the concentration-dependent inhibition of enzyme activity. At 1 mg·mL^−1^ of peel with hot water (81.84 mg·mL^−1^) and ethanolic (87.48 mg·mL^−1^) extracts, there was no significant difference in the *α*-glucosidase inhibition when compared to acarbose (83.94 mg·mL^−1^). All peel extracts showed the most increasing rate of inhibition ranging from 29.50 to 87.48 mg·mL^−1^ with significant differences. Meanwhile, the hot water extract of whole fruit showed the highest inhibition than others but was not significantly different from acarbose at a concentration of 0.05 mg·mL^−1^. However, pulp with hot water and ethanolic extracts showed the lowest *α*-glucosidase inhibition with a significant difference. When increasing the concentration of extracts, the results revealed that the percentage of *α*-glucosidase inhibition increased in all extracts. Inhibition of *α*-glucosidase activity was calculated with the IC_50_ values (Table 4). Most extracts had a higher value of IC_50_ than acarbose. In addition, the IC_50_ values of GBP peel with hot water and ethanolic extracts were determined to be 0.164 and 0.100 mg·mL^−1^, respectively. The inhibitory effect of the ethanolic peel extract was not significantly different from that of acarbose (IC_50_ 0.108 mg·mL^−1^). From these results, the ethanolic peel extract exhibited the potential inhibition of *α*-glucosidase activity. Moreover, the *α*-glucosidase inhibition of the hot water peel extract (0.164 mg·mL^−1^) suggested that there were no significant differences between ethanolic peel, hot water peel extracts and acarbose. Natural inhibitors derived from dietary plants are beneficial because they have a reduced inhibitory activity against *α*-amylase and a higher inhibitory activity against *α*-glucosidase [25]. Moreover, they can be utilised for the safe and efficient treatment of postprandial hyperglycaemia.

### 3.4. GC-MS Analysis

The ethanol extract of peel was subjected to GC-MS analysis to identify its phytochemical constituents. GC-MS analysis showed the presence of fourteen compounds in the extract (Table 7). The chromatographic analysis indicated the presence of numerous components in the ethanol peel extract that might be involved in biological processes. The maximum peak was shown by 9-octadecenamide (*Z*), followed by octadecanamide, hexadecanamide and 2,4-di-*tert*-butylphenol. Several of the compounds contained in the peel extract have already been demonstrated to possess antioxidant, antibacterial, hypocholesterolemic and antidiabetic characteristics [26, 27]. The compound 1-undecanol has previously been researched for its bactericidal and membrane-damaging properties [28]. Several publications have underlined the antioxidant and anti-inflammatory properties of 2,4-di-*tert*-butylphenol. Most significantly, the phenol displayed a wide range of toxicity in all tested species, including the producers, comprising cytotoxicity in human cells and animals, insecticidal and nematicidal activity, antibacterial activity and phytotoxicity [29]. 1-Hexadecanol is a 16-C fatty alcohol that can protect the skin against irritations triggered by insect bites, rashes and stings. It has been found that 1-hexadecanol inhibits the development of *Mycoplasma gallisepticum* and *Mycoplasma pneumoniae* [30], while 1-nonadecene has antitubercular, anticancer, antioxidant and antibacterial properties [28, 31]. *n*-Hexadecanoic acid demonstrates potential pharmacological activities including antioxidant, anti-inflammatory, anticancer, antitumour, hypocholesterolemic, nematicide and pesticide [32–35]. Decanoic acid has been demonstrated to possess substantial antibacterial and antifungal properties, and its esters have been employed in the medicinal, nutritional and dietetic fields [36]. Butyrate and its derivatives have the potential to serve as effective antibacterial and immunomodulatory therapeutics in the treatment of bacterial infections [37]. Hexadecanamide is a saturated fatty acid amide produced from palmitic acid. It has been investigated for potential therapeutic applications, such as its capacity to modulate inflammation, decrease oxidative stress and enhance cognitive performance [38]. Moreover, 9-octadecenamide (*Z*) is abundant in natural volatile oil with strong antioxidant, antibacterial, anti-inflammatory, antifungal and hypolipidaemic properties [39, 40].

### 3.5. Cytotoxic Activity

hGFs are accountable for a substantial portion of the extracellular matrix production in connective tissue and significantly impact wound healing [21]. GBP could be considered a functional food and has fewer side effects in comparison with conventional drugs, offering a safer alternative for the management of diabetes [5, 22]. Therefore, it is essential to verify the safety and effectiveness of GBP before their utilisation. In this respect, the cytotoxic potential of the GBP peel extract was assessed on hGF cells in comparison with SLS as a positive control. The results are presented in Table 8. hGF cells were treated with different concentrations of ethanolic peel extract (0.0001 to 1 mg·mL^−1^) to determine cell survival. The results showed that cell survival was 50% at 0.086 mg·mL^−1^ when cells were treated with SLS. However, when increasing the concentration of the peel extract to 1.0 mg/mL, hGF cells survived (87.62 mg·mL^−1^). The results indicated that the ethanolic GBP peel extract exerts cytotoxic effects on hGF cells with lower potency compared to SLS.

## 4. Conclusion

GBP has the potential to serve as a significant component in health food products. GBP peel is a good source of minerals and fibre. Further, it effectively inhibits carbohydrate-hydrolysing enzymes. The ethanolic peel extract displayed the highest potency in inhibiting both *α*-amylase and *α*-glucosidase activities, suggesting that the solvent has the capability to extract the active constituents present in GBP. GC-MS analysis indicated the presence of numerous components in the ethanolic peel extract that might be involved in biological processes. GBP peel appears to be a promising candidate that might be beneficial for various applications in plant-based superfoods, nutraceuticals and alternative medicine.

## Figures and Tables

**Table 1:** Proximate composition of GBP.

**Parts of GBP**	**Proximate composition**					
	**Ash (%)**	**Moisture (%)**	**Carbohydrate (%)**	**Fat (%)**	**Protein (%)**	**Total dietary fibre (%)**
Pulp	3.03 ± 0.21^c^	13.08 ± 0.42^a^	81.50 ± 0.22^a^	0.72 ± 0.06^c^	1.66 ± 0.07^b^	10.58 ± 0.56^c^
Peel	10.05 ± 0.52^a^	11.58 ± 0.38^b^	71.90 ± 0.04^b^	2.83 ± 0.11^a^	3.64 ± 0.29^a^	36.62 ± 1.41^a^
Whole fruit	3.80 ± 0.12^b^	11.30 ± 0.67^b^	81.79 ± 0.55^a^	1.09 ± 0.14^b^	2.01 ± 0.16^b^	13.49 ± 0.53^b^

*Note:* Each value is presented as mean ± SD (*n* = 3). Mean with different letters (a–c) shows statistically significant differences (*p* ≤ 0.05).

**Table 2:** *α*-Amylase inhibition effect from hot water GBP extracts at different concentrations (0.25–2.0 mg·mL^−1^).

**GBP extract concentration (mg·mL^−1^)**	***α*-Amylase inhibition (%)**			
	**0.25**	**0.5**	**1.0**	**2.0**
Acarbose	48.50 ± 3.10^Ac^	52.97 ± 1.81^Ab^	59.54 ± 1.54^Aa^	63.07 ± 0.81^Aa^
Pulp	38.54 ± 3.69^Bb^	41.01 ± 3.97^Bb^	44.18 ± 2.81^Cb^	53.72 ± 1.39^Ba^
Peel	35.92 ± 2.06^Bb^	38.13 ± 8.92^Bb^	44.44 ± 3.78^Cb^	54.02 ± 1.00^Ba^
Whole fruit	41.78 ± 3.35^Bb^	44.13 ± 4.62^ABb^	50.74 ± 0.49^Ba^	53.08 ± 2.27^Ba^

*Note:* Each value is presented as mean ± SD (*n* = 3). Mean within columns with different letters (A–C) shows statistically significant differences (*p* ≤ 0.05). Mean within rows with different letters (a–c) shows statistically significant differences (*p* ≤ 0.05).

**Table 3:** *α*-Amylase inhibition effect from ethanolic GBP extracts at different concentrations (0.25–2.0 mg·mL^−1^).

**GBP extract concentration (mg·mL^−1^)**	***α*-Amylase inhibition (%)**			
	**0.25**	**0.5**	**1.0**	**2.0**
Acarbose	48.50 ± 3.10^Ac^	52.97 ± 1.81^Ab^	59.54 ± 1.54^Aa^	63.07 ± 0.81^Aa^
Pulp	39.49 ± 1.59^Bc^	45.67 ± 3.60^Bb^	46.28 ± 1.39^Cb^	62.04 ± 1.22^ABa^
Peel	47.76 ± 0.95^Ac^	49.22 ± 2.28^ABbc^	53.08 ± 2.98^Bb^	58.69 ± 3.39^BCa^
Whole fruit	44.79 ± 1.13^Ac^	48.33 ± 2.57^ABbc^	51.33 ± 1.74^Bb^	55.76 ± 2.23^Ca^

*Note:* Each value is presented as mean ± SD (*n* = 3). Mean within columns with different letters (A–C) shows statistically significant differences (*p* ≤ 0.05). Mean within rows with different letters (a–c) shows statistically significant differences (*p* ≤ 0.05).

**Table 4:** IC_50_ (mg·mL^−1^) value from GBP with hot water and ethanolic extracts for *α*-amylase and *α*-glucosidase inhibition.

**Parts of GBP**	**IC_50_ (mg·mL^−1^)**	
	***α*-Amylase inhibition**	***α*-Glucosidase inhibition**
Acarbose	0.219 ± 0.08^e^	0.108 ± 0.025^d^

*Hot water extraction*		
Pulp	1.615 ± 0.173^a^	0.939 ± 0.023^b^
Peel	1.574 ± 0.128^a^	0.164 ± 0.006^cd^
Whole fruit	1.302 ± 0.359^ab^	0.403 ± 0.162^c^

*Ethanol extraction*		
Pulp	1.069 ± 0.07^bc^	1.294 ± 0.297^a^
Peel	0.512 ± 0.182^de^	0.100 ± 0.008^d^
Whole fruit	0.741 ± 0.240^cd^	0.219 ± 0.070^cd^

*Note:* Each value is presented as mean ± SD (*n* = 3). Mean with different letters (a–e) shows statistically significant differences (*p* ≤ 0.05).

**Table 5:** *α*-Glucosidase inhibition effect from hot water GBP extracts at different concentrations (0.05–1.0 mg·mL^−1^).

**GBP extract concentration (mg·mL^−1^)**	***α*-Glucosidase inhibition (%)**			
	**0.05**	**0.25**	**0.5**	**1**
Acarbose	38.23 ± 3.85^Ac^	60.54 ± 3.90^Ab^	79.75 ± 2.48^Aa^	83.94 ± 5.04^Aa^
Pulp	14.75 ± 1.26^Cc^	16.05 ± 0.35^Cc^	25.44 ± 5.62^Cb^	55.62 ± 0.77^Ba^
Peel	29.50 ± 1.42^Bd^	53.50 ± 2.13^Bc^	74.05 ± 3.82^Ab^	81.84 ± 1.28^Aa^
Whole fruit	41.10 ± 5.37^Ac^	47.58 ± 5.49^Bbc^	52.08 ± 2.45^Bb^	62.50 ± 5.54^Ba^

*Note:* Each value is presented as mean ± SD (*n* = 3). Mean within columns with different letters (A–C) shows statistically significant differences (*p* ≤ 0.05). Mean within rows with different letters (a–d) shows statistically significant differences (*p* ≤ 0.05).

**Table 6:** *α*-Glucosidase inhibition effect from ethanolic GBP extracts at different concentrations (0.05–1.0 mg·mL^−1^).

**GBP extract concentration (mg·mL^−1^)**	***α*-Glucosidase inhibition (%)**			
	**0.05**	**0.25**	**0.5**	**1**
Acarbose	38.23 ± 3.85^Ac^	60.54 ± 3.90^Ab^	79.75 ± 2.48^Aa^	83.94 ± 5.04^Aa^
Pulp	5.62 ± 2.14^Cd^	13.20 ± 2.19^Cc^	29.45 ± 2.61^Cb^	57.21 ± 5.63^Ca^
Peel	36.96 ± 1.10^Ad^	65.08 ± 1.11^Ac^	75.84 ± 2.33^Ab^	87.48 ± 1.50^Aa^
Whole fruit	29.14 ± 4.96^Bc^	53.72 ± 2.27^Bb^	60.09 ± 9.30^Bb^	74.08 ± 4.64^Ba^

*Note:* Each value is presented as mean ± SD (*n* = 3). Mean within columns with different letters (A–C) shows statistically significant differences (*p* ≤ 0.05). Mean within rows with different letters (a–d) shows statistically significant differences (*p* ≤ 0.05).

**Table 7:** Chemical profile of the ethanolic GBP peel extract by GC-MS.

**Peak**	**Retention time (min)**	**Compounds**	**Molecular weight**	**Chemical formula**
1	3.275	Ethane	118	C_6_H_14_O_2_
2	7.185	Tetraethyl silicate	208	C_8_H_20_O_4_Si
3	9.323	2-Azacyclooctanone	127	C_7_H_13_NO
4	13.486	1-Undecanol	172	C_11_H_24_O
5	14.988	2,4-Di-*tert*-butylphenol	206	C_14_H_22_O
6	15.986	1-Hexadecanol	242	C_16_H_34_O
7	18.229	1-Nonadecene	266	C_19_H_38_
8	19.928	*n*-Hexadecanoic acid	256	C_16_H_32_O_2_
9	20.230	Decanoic acid, ethyl ester	200	C_12_H_24_O_2_
10	20.286	Butanoic acid	320	C_20_H_32_O_3_
11	21.815	9,9-Dimethoxybicyclo[3.3.1]nona-2,4-dione	212	C_11_H_16_O_4_
12	21.990	Hexadecanamide	255	C_16_H_33_NO
13	23.570	9-Octadecenamide (*Z*)	281	C_18_H_35_NO
14	23.772	Octadecanamide	283	C_18_H_37_NO

**Table 8:** Percentage of survival of human gingival fibroblast cells treated with sulphorhodamine B (SRB).

**Samples**	**hGF cell survival (%)**				
Concentration (mg·mL^−1^)	0.0001	0.001	0.01	0.1	1.0
GBP peel extract	98.82 ± 1.39^a^	95.46 ± 1.83^a^	92.89 ± 2.46^a^	89.97 ± 6.35^a^	87.62 ± 3.85^a^
Sodium lauryl sulphate	95.82 ± 4.39^a^	92.15 ± 6.29^a^	84.30 ± 2.42^b^	43.17 ± 4.73^b^	17.21 ± 1.44^b^

*Note:* Each value is presented as mean ± SD (*n* = 4). Mean within column with different letters (a-b) shows statistically significant differences (*p* ≤ 0.05).

## Data Availability

The data used to support the findings of this study are included within the article.
